# Isoproterenol induced cardiac hypertrophy: A comparison of three doses and two delivery methods in C57BL/6J mice

**DOI:** 10.1371/journal.pone.0307467

**Published:** 2024-07-22

**Authors:** Patricia Perez-Bonilla, Brianna LaViolette, Bidur Bhandary, Soumya Ullas, Xian Chen, Dinesh Hirenallur-Shanthappa

**Affiliations:** 1 Global Discovery, Investigative & Translational Sciences–Animal Models and Imaging, Pfizer Inc, Cambridge, Massachusetts, United States of America; 2 Rare Diseases Research Unit, Pfizer Inc, Cambridge, Massachusetts, United States of America; The Open University, UNITED KINGDOM OF GREAT BRITAIN AND NORTHERN IRELAND

## Abstract

Heart Failure (HF) continues to be a complex public health issue with increasing world population prevalence. Although overall mortality has decreased for HF and hypertrophic cardiomyopathy (HCM), a precursor for HF, their prevalence continues to increase annually. Because the etiology of HF and HCM is heterogeneous, it has been difficult to identify novel therapies to combat these diseases. Isoproterenol (ISP), a non-selective β-adrenoreceptor agonist, is commonly used to induce cardiotoxicity and cause acute and chronic HCM and HF in mice. However, the variability in dose and duration of ISP treatment used in studies has made it difficult to determine the optimal combination of ISP dose and delivery method to develop a reliable ISP-induced mouse model for disease. Here we examined cardiac effects induced by ISP via subcutaneous (SQ) and SQ-minipump (SMP) infusions across 3 doses (2, 4, and 10mg/kg/day) over 2 weeks to determine whether SQ and SMP ISP delivery induced comparable disease severity in C57BL/6J mice. To assess disease, we measured body and heart weight, surface electrocardiogram (ECG), and echocardiography recordings. We found all 3 ISP doses comparably increase heart weight, but these increases are more pronounced when ISP was administered via SMP. We also found that the combination of ISP treatment and delivery method induces contrasting heart rate, RR interval, and R and S amplitudes that may place SMP treated mice at higher risk for sustained disease burden. Mice treated via SMP also had increased heart wall thickness and LV Mass, but mice treated via SQ showed greater increase in gene markers for hypertrophy and fibrosis. Overall, these data suggest that at 2 weeks, mice treated with 2, 4, or 10mg/kg/day ISP via SQ and SMP routes cause similar pathological heart phenotypes but highlight the importance of drug delivery method to induce differing disease pathways.

## Introduction

Heart Failure (HF) affects millions of people worldwide, and in the US, it constitutes a considerable and increasing amount of healthcare costs [1–3]. HF, which is typically preceded by hypertrophic cardiomyopathy (HCM), accounts for a substantial number of inpatient and outpatient in clinical settings annually [4–8]. Although advances in biomedical research have decreased mortality, the prevalence of HCM and HF has continued to increase [2, 4, 9–11]. HCM is characterized by a chronic physiological increase in cardiac muscle mass that results from cardiac wall stress [12]. Cardiac hypertrophy can be developed transiently, often as a compensatory mechanism during periods of sustained stress [12]. However, if the condition persists, hypertrophy leads to pathological remodeling of cardiac structures and ultimately HF [13, 14].

Several animal models of HCM and HF have been developed through a combination of genetic modifications, administration of pharmacologic compounds, and/or surgical approaches to recapitulate human disease [15–19]. One of the commonly used drugs to induce cardiotoxicity and develop disease in animal models is isoproterenol (ISP). ISP is a non-selective β-adrenoreceptor agonist, and as such its effects include increasing heart rate and stimulating mechanisms of oxidative stress, the renin-angiotensin-aldosterone system, and fibrogenic factors [20–29]. Notably, ISP is used alongside genetic modifications to induce disease at an earlier age or accelerate disease progression [30, 31]. ISP induces differing structural changes in the heart depending on dose, route, and duration of administration [20, 21, 29, 32]. Acute models of disease induce cardiac stress by using one or multiple bolus intraperitoneal or subcutaneous (SQ) ISP injections, while chronic models use implanted minipumps to release ISP continuously [5, 21, 27, 29, 32]. For example, β-adrenergic stimulation by ISP for more than 3 days results in cardiac hypertrophy [29], and acute ISP administration produces tachycardia associated with relative ischemia due to imbalance between increased myocardial oxygen demand and reduced coronary blood supply [33–35]. Thus, a principal advantage of using ISP is that acute and chronic cardiac injury can be induced by adjusting the dose and duration of administration, which leads to development of pathology from mild HCM to congestive HF [20].

In addition to dose and duration of ISP treatment, other considerations when developing ISP driven HF models are route of administration and delivery method. The route by which a compound is administered, can affect its bioavailability, distribution, metabolism, and pharmacological effect on the heart and other organs [21, 36–39]. The delivery, in turn, can modulate absorption rate, steady state concentration of the compound, and risk of complications. In rodents, ISP is commonly administered through a SQ or intraperitoneal (*i*.*p*.) route. However, ISP is delivered via daily injections or mini-pump infusions [5, 21, 27, 29, 32]. While previous studies compare exposure to ISP via distinct routes of administration in rodents, it remains unclear whether the extent of cardiac remodeling and dysfunction following ISP administration differs between delivery methods in C57BL/6J (B6-J) mouse models. In summary, variability in dose, treatment duration, route of administration, and delivery method has hindered efforts to develop reproducible mouse models of ISP driven HCM and HF. Thus, establishing an optimal dose and delivery system in combination with the use of genetic modifications is crucial to the development of translationally relevant mouse models of cardiovascular disease. Here, we sought to 1) determine a low ISP dose (2, 4, and 10mg/kg/day) at which myocardial injury and dysfunctional effects occur, and 2) assess whether daily subcutaneous (SQ) injections and SQ-minipump (SMP) infusions induce comparable disease severity in B6-J mice.

## Results

### Isoproterenol induces contrasting ECG parameters that are modulated by delivery method

**Fig 1:**
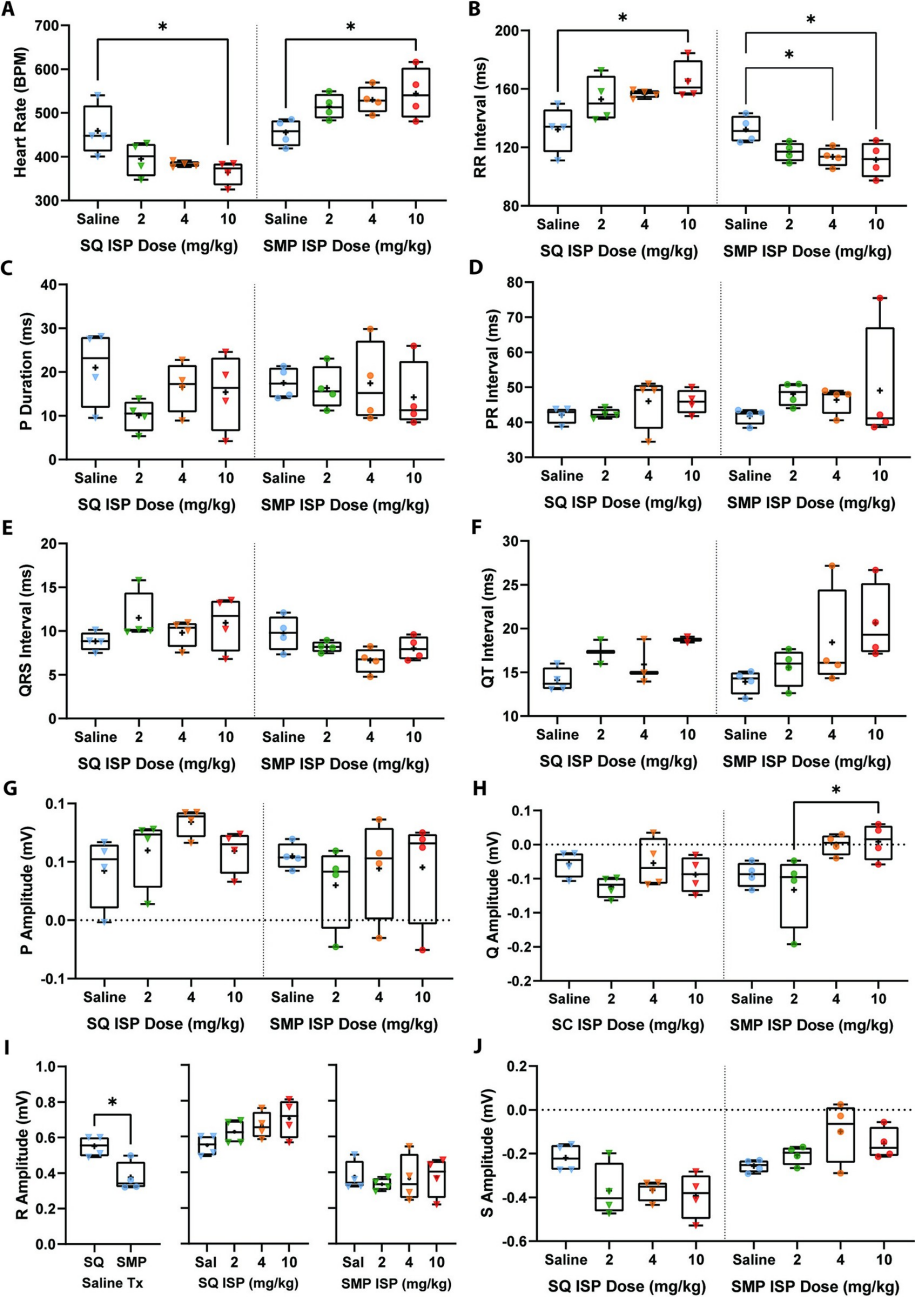
Surface ECG metrics for mice in SQ and SMP groups treated with ISP. For all graphs, both SQ and SMP treated groups are presented in the same X axis but are separated by a dotted line. SQ group is on left side and SMP group is on right side of dotted line. Symbols represent number of mice in each cohort (n = 4). A) heart rate, B) RR interval, C) PR interval, D) P duration, E) QRS interval, F) QT interval, G) P amplitude, H) Q amplitude, I) R amplitude, and J) S amplitude. Each boxplot represents min to max showing all points, line at median and plus sign at mean. RR interval, heart rate, P duration, Q, R and S amplitude data were analyzed using ordinary one-way ANOVA with Sidak’s multiple comparison’s test. PR, QRS, and QT interval, and P amplitude data were analyzed using Kruskal-Wallis ANOVA with Dunn’s multiple comparison’s test. * = *p*<0.05, ** = *p*<0.01, *** = *p*<0.001, **** = *p*<0.0001.

To determine whether delivery method, ISP dose, or the combination of both altered the pattern of electrical activity in the hearts of both SQ and SMP treated groups, anesthetized mice were placed on a warm pad with subcutaneous electrodes in a 3-lead ECG configuration. First, we found that the saline-treated SQ group was not significantly different to the saline-treated SMP group for most ECG measures except for R amplitude (Fig 1A–1J and S1 Table). Then, we examined the effect of ISP doses via each delivery method (SQ and SMP treated groups divided by dotted line). Heart rate (HR) was modestly decreased compared to saline in the SQ group by 2 and 4 mg/kg of ISP (ns trend), and significantly decreased by 10mg/kg of ISP (Fig 1A, Left). ISP treatment via SMP, however, induced a modest HR increase at 2 and 4 mg/kg (ns trend), and significantly increased HR at the 10 mg/kg dose (Fig 1A, Right). Thus, HR is dose dependently decreased by ISP in the SQ group, but dose dependently increased by ISP in the SMP group. When comparing the combination of dose and mode of delivery between groups, we found that for all doses, the SMP group had significantly higher HRs compared to the SQ group (S1 Table). Concomitant with HR dynamics, the RR interval was moderately lengthened in the SQ group compared to saline by 2 and 4 mg/kg of ISP (ns trend), and significantly increased by 10 mg/kg of ISP (Fig 1B, Left). In the SMP group, 2 mg/kg of ISP modestly shortened RR interval duration (ns trend), and significantly decreased RR interval duration at 4 and 10 mg/kg of ISP (Fig 1B, Right). In addition, when treated with either 2, 4 or 10mg/kg of ISP, SMP groups exhibit significantly shorter RR intervals when compared to SQ groups (S1 Table). P duration, and PR, QRS, and QT intervals were not altered by ISP doses in either the SQ or SMP groups when compared to respective saline controls, or to alternative delivery method groups (Fig 1C–1F and S1 Table). P amplitude was also unaltered by ISP dose and delivery method within and outside delivery method group (Fig 1G and S1 Table). Q wave amplitude is significantly increased at 10 mg/kg of ISP when compared to 2 mg/kg (Fig 1H) in SMP groups, suggesting that higher ISP doses induce greater ventricular contractions. R and S wave amplitudes are not significantly changed by all 3 doses of ISP when compared to their respective saline controls in both SQ and SMP treated mice (Fig 1I and 1J). However, mice in the SMP group treated with all ISP doses had significantly shorter R wave amplitude when compared to the respective dose groups treated via SQ daily injections (S1 Table). This trend was repeated with S amplitude, but only at 4 and 10 mg/kg of ISP. These results show that all three doses of ISP induce contrasting HR, RR Interval duration, and R and S amplitude that may be modulated by delivery method. Representative waterfall plots of the full recording period show overall changes in ECG waves for each dose (S1 Fig). Overall, these data indicate that delivery method, particularly methods that require surgery, can modestly modulate ECG baseline measures within 14 days of procedure, and that the combination of either SQ daily injections or SMP infusions and ISP dose induces a significant opposing effect that is evident in HR, RR interval, and R and S amplitudes.

### Isoproterenol treatment increases heart wall thickness and subcutaneous mini-pump implanted mice develop hypertrophic hearts

**Fig 2:**
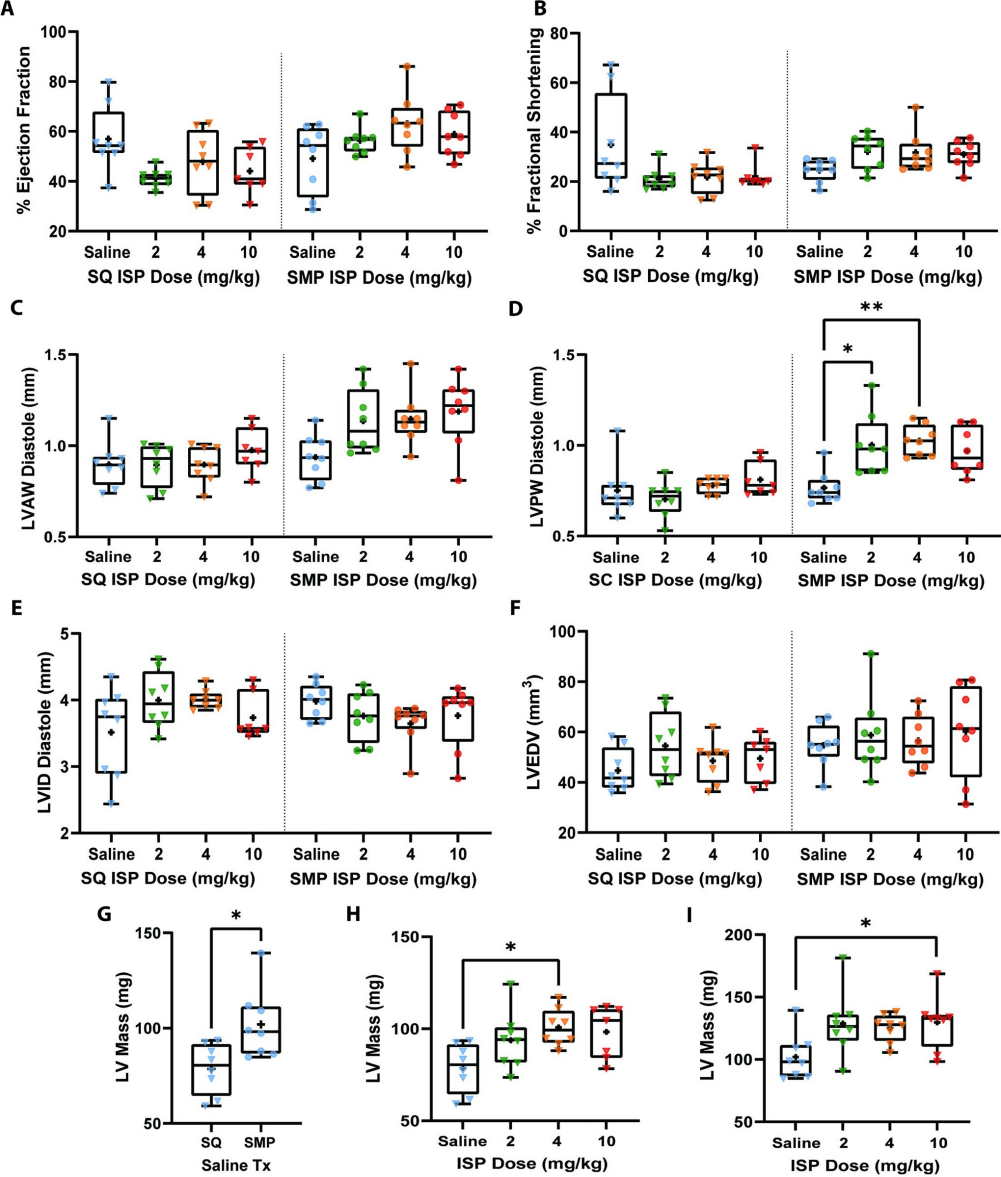
Heart function assessed via echocardiography. For all graphs, symbols represent the number of mice in each cohort (n = 8). A) % ejection fraction, B) % fractional shortening, C) diastolic left ventricular anterior wall thickness, D) diastolic left ventricular posterior wall, E) diastolic left ventricular internal diameter, F) left ventricular end diastolic volume, G) left ventricular mass, saline treated mice only, F) left ventricular mass SQ only, and G) left ventricular mass SMP only. In A-F, both SQ and SMP treated groups are presented in the same X axis but are separated by a dotted line. SQ group is on left side of dotted line and SMP group is on right side. Each boxplot represents min to max showing all points, line at median and plus sign at mean. Data in A, C, E-G were analyzed using ordinary one-way ANOVA with Sidak’s multiple comparison’s test and data in B and D were analyzed using Kruskal-Wallis ANOVA with Dunn’s multiple comparison’s test. * = *p*<0.05, ** = *p*<0.01, *** = *p*<0.001, **** = *p*<0.0001.

We next examined whether delivery method, ISP dose, or a combination of both, modified heart function through echocardiography (Fig 2A–2I and S2 Table, S1 and S2 Figs). When comparing SQ and SMP mice treated with saline alone, we found no significant differences in ejection fraction, fractional shortening, left ventricular anterior and posterior wall thickness at diastole, left ventricular internal diameter at diastole, and left ventricular end diastolic volume (Fig 2A–2E and S2 Table). However, mice treated with saline via SMP infusion had significantly higher left ventricular mass compared to mice treated with saline via SQ injection (Fig 2G and S2 Table). These data suggest that 2 weeks post-surgery, mice treated via SMPs may be at higher risk for cardiac dysfunction at baseline than mice being treated via SQ injections.

To determine the effect of ISP on cardiac function via different delivery methods, we then evaluated mice treated with increasing doses of ISP (Fig 2A–2F and 2H, 2I). Within SQ or SMP groups (divided by dotted line), all 3 ISP doses did not impact ejection fraction, fractional shortening, left ventricular anterior wall thickness at diastole, left ventricular internal diameter at diastole, or left ventricular end diastolic volume when compared to respective saline controls (Fig 2A–2C and 2E, 2F). However, we found that left ventricular posterior wall thickness at diastole was significantly increased in SMP group treated with 2 and 4 mg/kg of ISP (Fig 2D, Right), and that at 10 mg/kg of ISP, left ventricular mass was also increased for SMP group (Fig 2I). In the SQ treated group, we found a significant increase in LV Mass when mice were treated with 4 mg/kg of ISP (Fig 2H). These results suggest that 2 weeks of ISP doses up to 10 mg/kg via both SQ and SMP delivery is not sufficient to robustly impact functional myocardial dynamics.

Lastly, SQ and SMP groups significantly differ at specific ISP doses; ejection fraction across all doses, fractional shortening at 2 and 10 mg/kg, left ventricular anterior wall thickness across all doses, and left ventricular posterior wall thickness at 2 and 4 mg/kg (S2 Table). Taken together, these data suggest that at 2 weeks, mice treated with ISP doses up to 10 mg/kg via SMP may experience more detrimental cardiac phenotypes when compared to SQ treated mice.

### Subcutaneous mini-pump implanted mice have greater body, lung, and heart weights, and Isoproterenol treatment aggravates these effects

**Fig 3:**
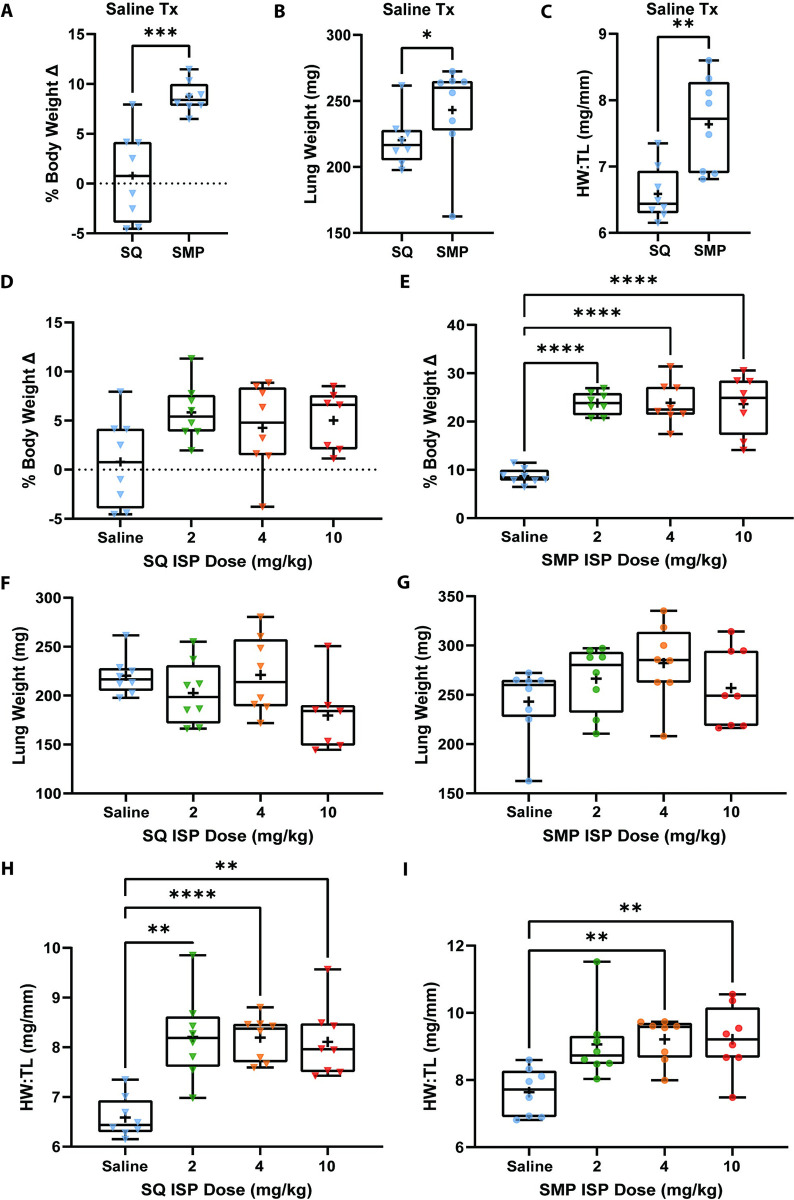
Body, lung, and heart weights. Top of figure contains legend. For A-I, symbols represent number of mice in each cohort (n = 8). A-C show saline treated mice only. A) % body weight change over 2 weeks, B) lung weight, C) heart weight over tibia length. Each boxplot represents min to max showing all points, line at median and plus sign at mean. Data were analyzed with unpaired t-test. D-I show saline, 2, 4, or 10mg/kg ISP treated mice. On the left, SQ treated mice and on the right, SMP treated mice. D) SQ group % body weight change, E) SMP group % body weight change, F) SQ group lung weight, G) SMP group lung change, H) SQ group heart weight over tibia length, and I) SMP group heart weight over tibia length. Each boxplot represents min to max showing all points, line at median and plus sign at mean. Data were analyzed using ordinary one-way ANOVA with Sidak’s multiple comparison’s test. * = *p*<0.05, ** = *p*<0.01, *** = *p*<0.001, **** = *p*<0.0001.

We then examined whether delivery method alone altered body, lung, and heart weight while mice were being treated with saline via daily SQ injections or SMP infusions (Fig 3A–3C). We found that % body, lung, and heart weight is significantly increased in mice treated via SMP (Fig 3A–3C), suggesting that SMP implanted mice may have higher disease burden at baseline compared to SQ treated mice. Since delivery method induced significant changes in % body, lung, and heart weight, we next assessed response to ISP doses within delivery method groups separately. We found that % body weight did not change for mice treated via daily SQ injections when compared to saline (Fig 3D) but was further increased from saline with all 3 ISP doses in the SMP group (Fig 3E). In addition, the magnitude of body weight change compared to saline treated mice was greater for SMP treated mice. Lung weight under all 3 ISP doses was not significantly different from saline controls in both the SQ and SMP groups (Fig 3F and 3G). However, ISP treatment significantly increased heart weight for both SQ and SMP treated groups when compared to saline treated mice in the respective groups (Fig 3H and 3I). Taken together, these data suggest that changes in body, lung, and heart weight are modulated by delivery method, and that treatment with ISP exacerbates these alterations. After 2 weeks of treatment, SMP implanted mice showed an overall increase in body, lung, and heart weight that was equivalent for all 3 doses of ISP.

### Isoproterenol promotes expression of hypertrophic and fibrotic gene markers

**Fig 4:**
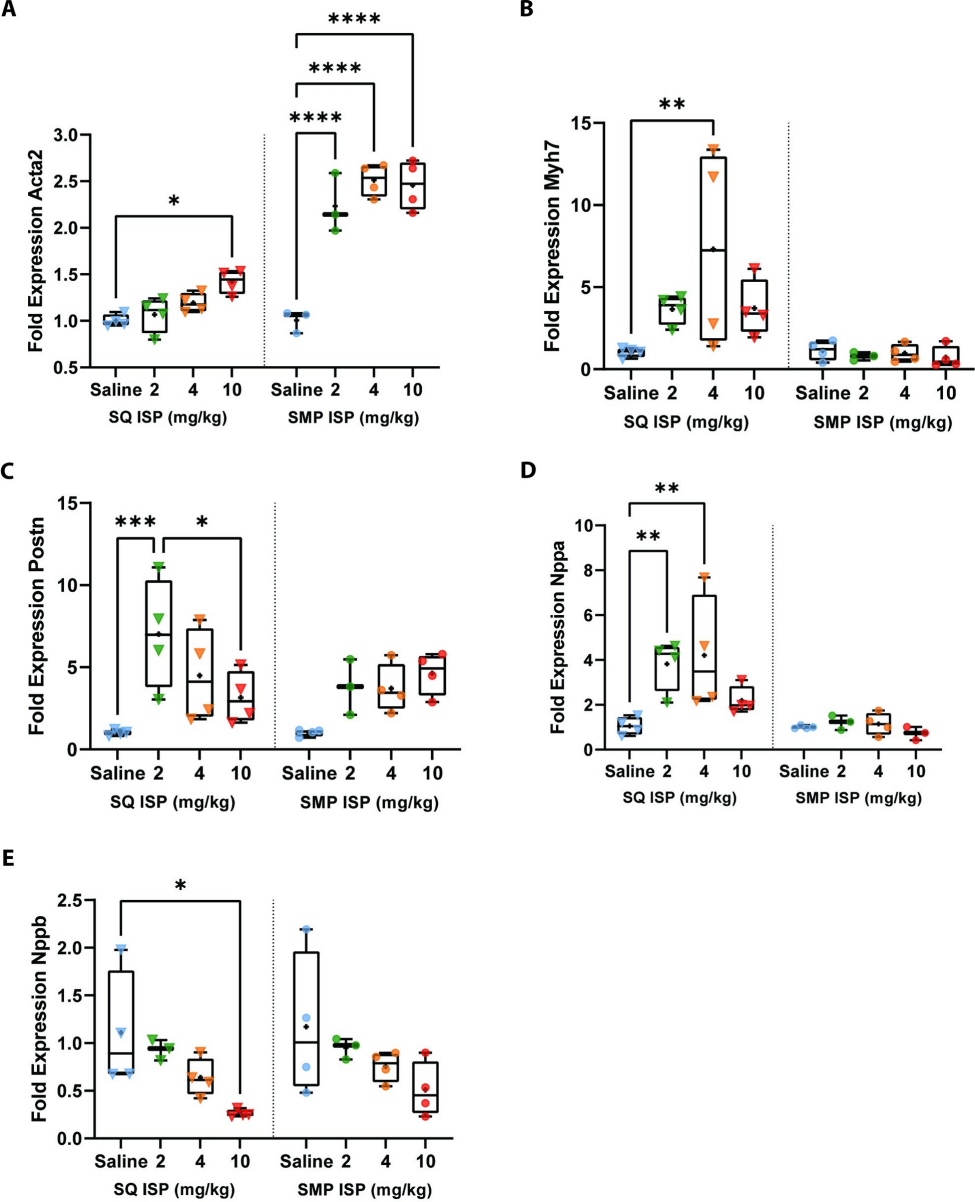
Gene expression changes after 2 weeks of ISP treatment via SQ and SMP. For A-E, symbols represent number of mice in each cohort (n = 3–4). On the left of dotted line, triangles represent mice treated via SQ and on the right of dotted line, circles represent mice treated via SMP. A) *Acta2*, B) *Myh7* C) *Postn*, D) *Nppa*, and E) *Nppb*. Each boxplot represents min to max showing all points, line at median and plus sign at mean. Data were analyzed using ordinary two-way ANOVA with Tukey’s multiple comparison’s test. * = *p*<0.05, ** = *p*<0.01, *** = *p*<0.001, **** = *p*<0.0001.

Lastly, we examined whether ISP and/or delivery method induced cellular markers of hypertrophy and fibrosis in the mouse myocardium. We found that delivery method alone had no significant effect on hypertrophic and fibrotic markers tested in heart tissue, as saline only comparison between SQ and SMP groups was not significantly different (Fig 4A–4E and S3 Table). Within SQ or SMP groups however (separated by dotted line), we found that ISP had a dose-dependent effect. Hypertrophic marker smooth muscle alpha-2 actin (*Acta2*) was significantly increased in the SQ group only at ISP 10mg/kg, but in the SMP group ISP increased *Acta2* fold expression at all 3 doses (Fig 4A). When examining beta-myosin heavy chain (*Myh7)*, another hypertrophic marker, we only observe an increase in the SQ group at ISP 4mg/kg, and a non-significant increase trend for the SQ group overall that we did not observe in the SMP group (Fig 4B). We also found that the hypertrophic marker periostin (*Postn*), is increased in SQ group at ISP 2mg/kg (Fig 4C, Left). In the SMP group, we found that ISP treatment increases *Postn* expression (ns trend), indicating that the combination of SMP and any of the 3 ISP doses induces fibrogenesis with 2 weeks of exposure (Fig 4C, Right). These results suggest that the combination of SMP and a low ISP dose promotes a more salient hypertrophic and fibrogenic state.

We then assessed cardiac stress by examining natriuretic peptide A (*Nppa*) and natriuretic peptide B (*Nppb*) in the ventricular myocardium and found that in the SQ treated group ISP at 2 and 4 mg/kg increased *Nppa* (Fig 4D, Left) and that ISP 10 mg/kg decreased *Nppb* (Fig 4E, Left). We found no significant differences in the SMP group for both *Nppa* and *Nppb* expression (Fig 4D and 4E, Right). These results suggest that in the SQ group, ISP daily injections are promoting maladaptive hypertrophy, fibrosis, and vascular remodeling. We also found that these effects are mediated solely by ISP, and not by delivery method (S3 Table). Taken together, these results confirm the previously reported effects of low dose ISP on inducing hypertrophy and fibrotic states, and these effects are independent from delivery method.

## Discussion

ISP is a non-selective β-adrenoreceptor agonist that is commonly used in rodent studies to induce acute and chronic HCM and exacerbate the development of HF [16–18]. However, the variability in both dose and route of administration reported in the literature has made it difficult to determine the optimal combination of ISP dose and delivery method to develop a reliable ISP induced injury mouse model. Notably, different mouse strains show significant differences in susceptibility to ISP and cardiac damage, thus confounding findings when ISP is used as a second hit alongside genetic manipulations to induce myocardial injury and create translationally relevant models of disease [40–43]. B6 mice on an N background, for example, are more susceptible to oxidative stress and cardiac injury compared to B6-J mice due to a mutation in the nicotinamide nucleotide transhydrogenase enzyme that results in ameliorated pressure-overload phenotypes in B6-J mice [44]. Since B6-J mice may have lower susceptibility to ISP, we established 2mg/kg as the lowest dose for this study. Here we administered 3 doses of ISP (2, 4, or 10mg/kg/day) over two weeks to demonstrate that male B6-J mice have a similar risk for cardiac injury via SQ and SMP delivery methods. Surprisingly, mice implanted with SMP showed decreased R wave amplitude and increased LV mass when given saline, suggesting that implanting a minipump to administer compounds can modulate physiological ECG and Echo responses at baseline. This may be due to altered systemic hemodynamics that are dependent on mode of delivery [32], as SMP surgery/implantation may elicit responses from the surrounding tissues that could alter normal physiological fluid balance that lead to edema as a secondary outcome. In addition, the combination of either SQ injections or SMP infusions and ISP dose induces a significant opposing effect that is evident in ECG measures. We also found that having a SMP promoted the development of hypertrophy, advanced enlargement of heart and lung tissue, and increased body weight. In addition, ISP treatment, at all 3 doses, additively aggravated the effects observed when mice were treated with saline only. Lastly, we found that ISP induced differential cardiac remodeling and expression of hypertrophic and fibrotic markers that were modulated by mode of delivery. Taken together, these data suggest that a low dose (up to 10 mg/kg) of ISP maintained via SQ and SMP for two weeks is sufficient to induce cardiac hypertrophy in C57BL/6J models, and that daily injections and continuous infusion of ISP into the SQ space are good models of intermittent and chronic β-adrenergic stimulation.

ISP is routinely used to induce myocardial injury and promote the development of HCM and HF in diverse mouse strains [42, 45, 46]. Indeed, a single high ISP dose, as is used in Takotsubo syndrome models, leads to the development of acute cardiac stress, severe cardiac dysfunction, and robust myocardial infiltration of macrophages, ischemia, and HF [20, 47, 48]. In their extensive review, Nichtova et al. (2012) classify the effects of ISP in the literature according to low, medium, and high doses of ISP in rodent models [20]. Since one of our goals was to identify a low ISP dose that could induce myocardial injury and cardiac disease in B6-J mice, we used ISP doses in the low-medium category according to this report. Here we found that when B6-J mice are exposed to two weeks of daily treatment, a low dose up to 10 mg/kg is sufficient to induce limited electrophysiological, functional, and molecular changes in the myocardium. These alterations, moreover, were modulated by delivery method.

In mice, ISP is commonly delivered into the SQ or *i*.*p*. space with either daily injections or minipump infusions [5, 21, 27, 29, 32]. In this study, SQ route was determined as the ideal route of administration, as it avoids the pharmacokinetics associated with enteral absorption, and allows for injection of larger volume fluids in small animals with minimal technical challenge [49]. Thus, both SQ and SMP groups received ISP into the SQ space, maintaining a consistent route of administration. However, because of the nature of these techniques, mice were exposed to ISP either intermittently (SQ) or continuously (SMP), altering delivery method.

Both intermittent and sustained patterns of β-adrenoreceptor stimulation contribute to the development of HF with varying degrees of cardiac remodeling [29, 50]. Daily ISP injections are described to mimic stress-induced cardiomyopathy, as ISP has a short half-life (2–5 minutes) and induces rapidly reversible acute left ventricular systolic dysfunction in the absence of heart disease [51–53]. In contrast, continuous infusion, and sustained exposure to ISP mimics advanced HF where there is chronic adrenergic stimulation. This approach is limited by the need for surgery expertise and infusion timeframe, as mini-pumps used here can continuously provide the studied compound for only 21 days. Both acute and chronic exposure to ISP are posited to induce receptor desensitization and downregulation [54]. Here, we found that B6 mice treated via SQ daily injections have decreased HR when compared to saline controls and SMP infused mice. An explanation for this is that we injected mice with ISP in the afternoon, but recorded ECGs in the following morning, thus we captured the response to ISP hours after bolus injection and period of circulation and metabolism. This result also suggests that administering ISP via bolus SQ injections may elicit receptor desensitization and downregulation as observed in a similar report [41]. As observed in other studies, however, SMP infusion of ISP increases and maintains HR at a higher level, also suggesting that continuous infusion of ISP may mask the initial compensatory adaptive response to the insult [29, 55]. As HR and RR interval are inversely correlated, we found the opposite effect on RR interval duration. We also found other typical characteristics of hypertrophy observed in ECGs from anesthetized rodents and isolated hearts such as longer QT duration and decreased R amplitude in SMP treated mice [56–58]. Overall, these results indicate that the combination of delivery method and low ISP dose influences the mechanism of disease development.

When examining cardiac function via echocardiography, we found that SQ and SMP ISP delivery elicited similar effects. Although there is no evident contractile impairment in mice treated via the two delivery methods, mice treated via SMP may show more significant morphological changes due to the additive development of increased heart wall thickness and mass in this timeframe. These alterations may be due to physiological hypertrophy with increased myocyte size, myocyte proliferation, connective tissue remodeling, or edema. However, our experimental design does not directly distinguish among these different modes of increased ventricular mass. These results suggest that administering ISP with different pharmacologic protocols could induce increases in ventricular weight by different stimuli that manifest with different molecular and cellular mechanisms [32]. Another explanation is that the responses elicited by ISP in 2 weeks are part of signaling pathways characteristic for adaptation [59]. Indeed, mice being treated with 10mg/kg of ISP via SMP for 26 days maintained cardiac function, indicating a possible cardioprotective effect during chronic activation of β-adrenergic receptors [55]. As expected, ISP up to 10 mg/kg increased heart weight in mice treated via both SQ and SMP. However, body weight and wet lung weight were increased in mice treated via SMP at baseline, suggesting the possibility of fluid accumulation as a response to the SMP implant and surgery insult. However, to conclusively determine peripheral and/or pulmonary edema in these mice, it would be best to measure inflammatory cytokines and the ratio of wet to dry lung in future studies.

Since cardiac hypertrophy is an adaptive response of the myocardium to pressure overload or adrenergic agonists, it is characterized by increased heart size, upregulated protein synthesis, induction of fetal gene program, and aberrant organization of the sarcomere structure [60]. In line with well documented ISP effects on cardiac gene expression, we assessed reactivation of the fetal program to find cardiac remodeling and expression of hypertrophic and fibrotic markers in both SQ and SMP treated mice with all doses. Characteristically, ISP induces upregulation of *Myh7*, *Acta2*, *Postn*, as well as ventricular expression of *Nppa* and *Nppb*. Upregulation of these gene markers indicate increases in cardiac stress, muscle contraction, and fibrosis. In mice treated via both SQ and SMP, *Acta2* was increased, but this increase was more pronounced in SMP treated mice. This may be due to the additive insult of ISP delivered continuously through the SMP. Interestingly, we did not observe this effect with other markers tested, however, we only found significant increases in gene expression of *MyH7*, *Postn*, *Nppa*, and *Nppb* in mice treated with ISP via SQ. Interestingly, a recent article explored the effect of 10mg/kg ISP withdrawal in B6-N mice after 26 days of continuous infusion via SMP to find that the induction of maladaptive cardiac remodeling mechanisms, including a switch to pathological gene expression, were counteracted by adaptive responses while ISP was being infused [55]. Notably, responses here may be limited due to the partial recapitulation of complex neurohumoral overactivation and cardiac remodeling mechanisms induced by ISP. Studies show that the combination of ISP and chronic angiotensin II or α-adrenergic stimulation with phenylephrine further mimic the pathophysiological sympathetic overdrive in HF, leading to more pronounced fibrotic phenotypes via increased cytokine release and pressure-overload driven early transcriptome alterations [26, 61–64]. These results are in line with studies showing that ISP only elicits mild pro-fibrotic effects in B6-J mice [41, 65], and suggest that mode of drug delivery may influence the signaling pathway and mechanism of disease development [29, 66].

Although the results here provide clarity on the intricacies of using low ISP doses, and the implications of using different modes of delivery, this study has limitations to consider. We only used male mice here, thus we cannot draw conclusions on the possibility of sex-related differences in the modulation of ISP dose response and mode of delivery. A recent study, however, reported lack of significant sex differences in ISP cardiac hypertrophy, dysfunction, and fibrosis in C57BL/6N mice, suggesting that female sex may not be sufficient to protect the heart against a severe pathologic stimulus and that sexual dimorphism in cardiovascular diseases is highly model-dependent [32, 67]. However, we used B6-J mice here, which may impact sex-related differences due to strain background. Of note, B6-J mice have a nicotinamide nucleotide transhydrogenase (Nnt) mutation that limits production of reactive oxygen species and ameliorates the development of pressure overload-induced heart failure [44]. This may explain why we did not find overt cardiac disease phenotypes here and highlights the need for more cardiac β-adrenergic receptor function studies in female preclinical models. We also consider the number of mice used here and the variability in data as limitations on statistical power. In addition, this study design only presents results from a single timepoint (2 weeks after ISP treatment) to measure disease, but remodeling is a progressive, dynamic response to injury that develops with time, and collecting data from further timepoints may have revealed more salient disease phenotypes in these mice [68]. Additionally, this study focused only on functional and physiological methods of assessment including ECG, echocardiography, and body and wet organ weights without delving into histopathological changes induced by ISO in the two groups, as these findings have been previously reported [69–73]. Previous studies also report cardiomyocyte changes in response to β-adrenergic agonism, confirming that acute and chronic adrenergic activation leads to oxidative stress, and cardiomyocyte injury and loss [24, 74–80]. Finally, while this study illustrated induction of significant myocardial dysfunction in both groups, there were differences between the two groups in some ECG, diastolic function, and molecular markers that this study was not sufficiently powered to establish.

In summary, this study demonstrates that in B6-J mice, both intermittent and sustained stimulation of β-adrenergic receptors for 2 weeks cause similar pathological phenotypes in the heart. One important aspect to consider for future studies is that although serial injections may be the preferred mode of administration due to being a less challenging technique that is also lower cost and permits better animal mobility [32], SMP implantation adds a layer of physiological complexity that may be necessary to mimic specific disease pathways. In this study, we found that mice with implanted SMPs showed greater body, lung, and heart weight, which may be due to changed hemodynamics, inflammation induced by surgery, and edema that was not present in SQ treated mice. Or findings significantly further our understanding of β-adrenergic stimulation in relation to cardiac pathophysiology, highlighting the importance of the mode of drug delivery and ISP dosage level.

## Materials and methods

### Mice

8–10-week-old male C57BL/6J mice were purchased from Jackson Labs (n = 64) and used for all experiments over the course of 30 days. Mice were maintained on Inotiv 2916 rodent diet (Teklad global 16% protein) and Innovive chlorinated water (M-WB-300C) and housed in a light (12hr light/ 12hr dark) and temperature (25°C) controlled environment. All procedures were carried out and approved in accordance with the Pfizer Institutional Animal Care and Use Committee (IACUC) regulations and established guidelines.

### Isoproterenol preparation and dosing

Mice were randomized by weight and classified into either a daily SQ injection (n = 32) or an SMP continuous infusion (n = 32) group. Within each group, mice were then randomly assigned to 1 of 8 dosing cohorts: SQ saline 10mL/kg, SMP saline 10mL/kg, SQ 2mg/kg, SMP 2mg/kg, SQ 4mg/kg, SMP 4mg/kg, SQ 10mg/kg or SMP 10mg/kg (n = 8 for each group). Confounders for animal cage/location, treatment order, and measurements were not evaluated. Mice were treated for 14 consecutive days. ISP SQ and SMP solutions were prepared by serially diluting ISP (Millipore Sigma, Sigma-Aldrich #16504) into sterile saline. ISP aliquots for SQ administration were covered from light and kept at -20°C until thawed before injection. SQ cohort received injections to the left flank at approximately the same time each day 3hrs before start of dark cycle. Prior to injection, the site between ribs and hind leg was cleaned with an alcohol swab, and a 26-gauge syringe was inserted into the SQ space at a 15–30-degree angle. Saline or ISP dose was injected steadily, and a 3–5 second pause after injection was implemented to prevent loss of compound.

### Body weights

Full body weights were measured a week before treatment start to permit randomization by weight into distinct groups. Full body weights were then taken again once on the day of first injection or surgery to obtain baseline measures, and 14 days later at end of study.

### Osmotic mini-pump implantation

Under sterile conditions, mice were subcutaneously implanted in the right flank with an osmotic mini-pump (Alzet Model 1002) for continuous infusion during the experiments. Isoflurane was used as anesthetic during implantation surgery. The skin incision (<1–1.25cm) was created over the left scapula region and closed with silk suture. A subcutaneous injection of carprofen in the form of MediGel CPF (Clear H2O or Meloxicam SR) and Buprenorphine were used as pain medication for pre-op and post-op care. Mice were recovered from surgery in an incubator in home cage and were removed from the incubator once ambulatory.

### Surface electrocardiography

Only 50% of mice were recorded for this experiment (SC n = 16, SMP n = 16). Mice were anesthetized with 3% isoflurane in induction chamber and maintained at 2% via nosecone. Extremities were secured with paper tape to a warming pad (VisualSonics Mouse Handling Platform 2, maintaining temperature at 37°C for the duration of recording), and needle electrodes (AD instruments 12mm long, 29 gauge) connected to a preamplifier (AD instruments Power Lab 16/35 and Bio amp) were inserted into the subcutaneous space in a lead II configuration. Sampling rate was 4k/s and heart rate was monitored throughout. Recording lasted for 5 min, last 2min were analyzed for graphs. Mice were recovered on a heated pad in home cage. Data was recorded in LabChart 8 and analyzed with LabChart Pro v8.1, where settings were manually established to reflect every beat (no averaging), Bazett QTc, typical QRS width of 10ms, R waves at least 60ms apart aligned to QRS maximum, a Pre-P baseline and maximums of 20, 80, and 60ms, and an ST height at 10ms from alignment.

### Echocardiography

Mice were under 1.5–2% sevoflurane anesthesia while recordings were taken with the Vevo 3100 Preclinical Imaging System (FUJIFILM VisualSonics Inc, Toronto, Canada). B-mode images from long axis and M-mode images from short axis were obtained, and data was analyzed using Graphpad Prism version 9.5.1 for Windows (GraphPad Software, San Diego, California USA).

*Tissue Collection*: After sublethal CO2 inhalation and cervical dislocation, heart, lung, and tibia were collected. Heart: ventricles were weighted and then left ventricle was immediately snap-frozen on liquid nitrogen and stored at -80˚C for molecular biology. Lungs were weighted and tibias measured for length and subsequently discarded.

### Gene expression

Heart tissue was dissected and immediately snap frozen on liquid nitrogen and stored at -80˚C. RNA was then extracted using Trizol (Invitrogen) and 200 ng samples were converted to cDNA using the Superscript First Strand Synthesis System for RT-PCR (Invitrogen). Sample cDNAs were analyzed in triplicate via quantitative RT-PCR for gene expression using TaqMan reagents and an ABI 7500 (Applied Biosystems). With GAPDH expression as an internal control, relative mRNA expression values were calculated by the 2-ΔΔCt method.

### Humane endpoint procedures

All mice were euthanized approximately 2 weeks after 1^st^ isoproterenol dose administration, unless early markers associated with death, poor prognosis of quality of life, or specific signs of distress were observed during daily monitoring. During surgery and recovery period, mice were provided with post-operative pre- and post-operative analgesia treatment as needed. Health condition was assessed based on breathing, body condition, activity level, hydration, wound healing, body weight. Monitoring personnel consulted with veterinary services if an animal was found abnormal in health status check and appropriate treatment was performed based veterinary services recommendations. One mouse in the SQ 10mg/kg group was euthanized before conclusion of the study due to health concern, data from this mouse was not included.

### Statistics

All values are presented as boxplots showing min to max and all points, line at median and plus sign at mean. Normally distributed data with equal standard deviation was analyzed with unpaired student’s t-test or ordinary one-way ANOVA with Sidak’s multiple comparison’s test. Abnormally distributed data, and data with unequal standard deviation was analyzed with unpaired Mann-Whitney test, Kruskal-Wallis ANOVA with Dunn’s multiple comparison’s tests, or Welch’s ANOVA with Dunnett’s T3 multiple comparison’s test. An alpha of 0.05 was used to determine statistical significance. * = *p*<0.05, ** = *p*<0.01, *** = *p*<0.001, **** = *p*<0.0001.

## Supporting information

S1 Fig Surface ECG waterfall plots. Each waterfall is a 3D plot of averaged beats over during 2min ECG recording. Each horizontal layer of the plot correspond to a full ECG cycle, with the earliest recordings at the top, and the latest at the bottom. Intermittent irregularities in the waves are indicators, but not determinants, of cardiac pathologies.
(TIF)

S2 Fig SQ echocardiography representative images. Echocardiography representative images for all SQ groups.
(TIF)

S3 Fig SMP echocardiography representative images. Echocardiography representative images for all SMP groups.
(TIF)

S1 Table Surface ECG statistics for comparison of mice in SQ vs SMP groups. Statistical tests used to compare between SQ and SMP groups treated with saline and all ISP doses showing mean and summary values.
(PDF)

S2 Table Heart function assessed via echocardiography comparison of mice in SQ vs SMP groups. Statistical tests used to compare between SQ and SMP groups treated with saline and all ISP doses showing mean and summary values.
(PDF)

S3 Table Gene expression changes comparison of mice in SQ vs SMP groups. Statistical tests used to compare between SQ and SMP groups treated with saline and all ISP doses showing mean and summary values.
(PDF)

S4 Table Raw data for all figures. Data behind means and averages represented in the manuscript.
(XLSX)
