# Supplementary figures and images for: Isoproterenol induced cardiac hypertrophy: A comparison of three doses and two delivery methods in C57BL/6J mice
Source: PLoS One. 2024 Jul 22;19(7):e0307467. doi: 10.1371/journal.pone.0307467 (PMC11262646; doi:10.1371/journal.pone.0307467)

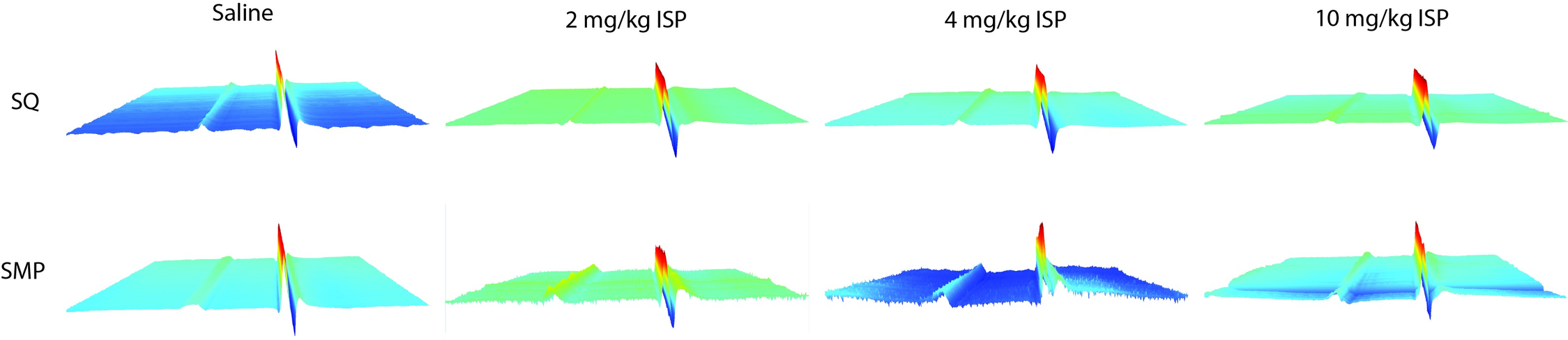

Supplement: S1 Fig — Each waterfall is a 3D plot of averaged beats over during 2min ECG recording. Each horizontal layer of the plot correspond to a full ECG cycle, with the earliest recordings at the top, and the latest at the bottom. Intermittent irregularities in the waves are indicators, but not determinants, of cardiac pathologies. (TIF) [file pone.0307467.s001.tif]

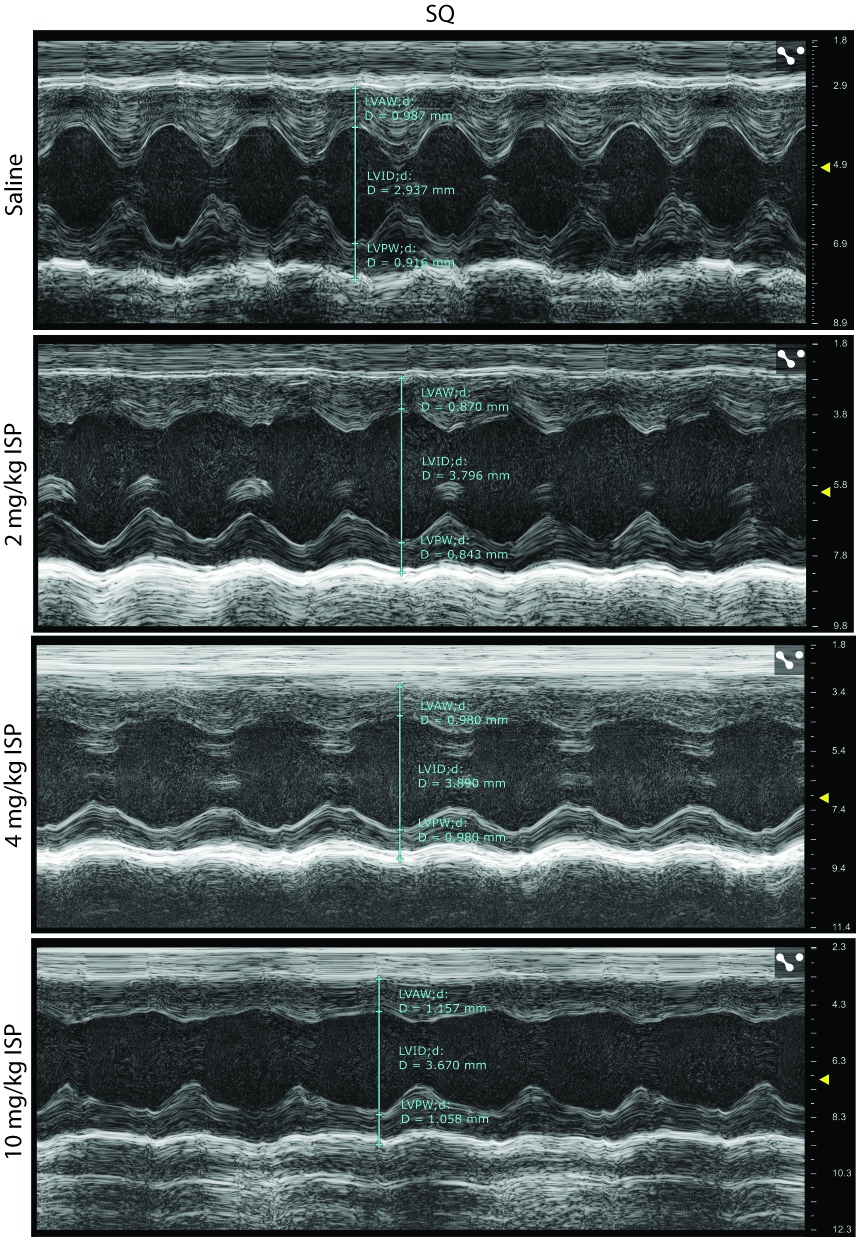

Supplement: S2 Fig — Echocardiography representative images for all SQ groups. (TIF) [file pone.0307467.s002.tif]

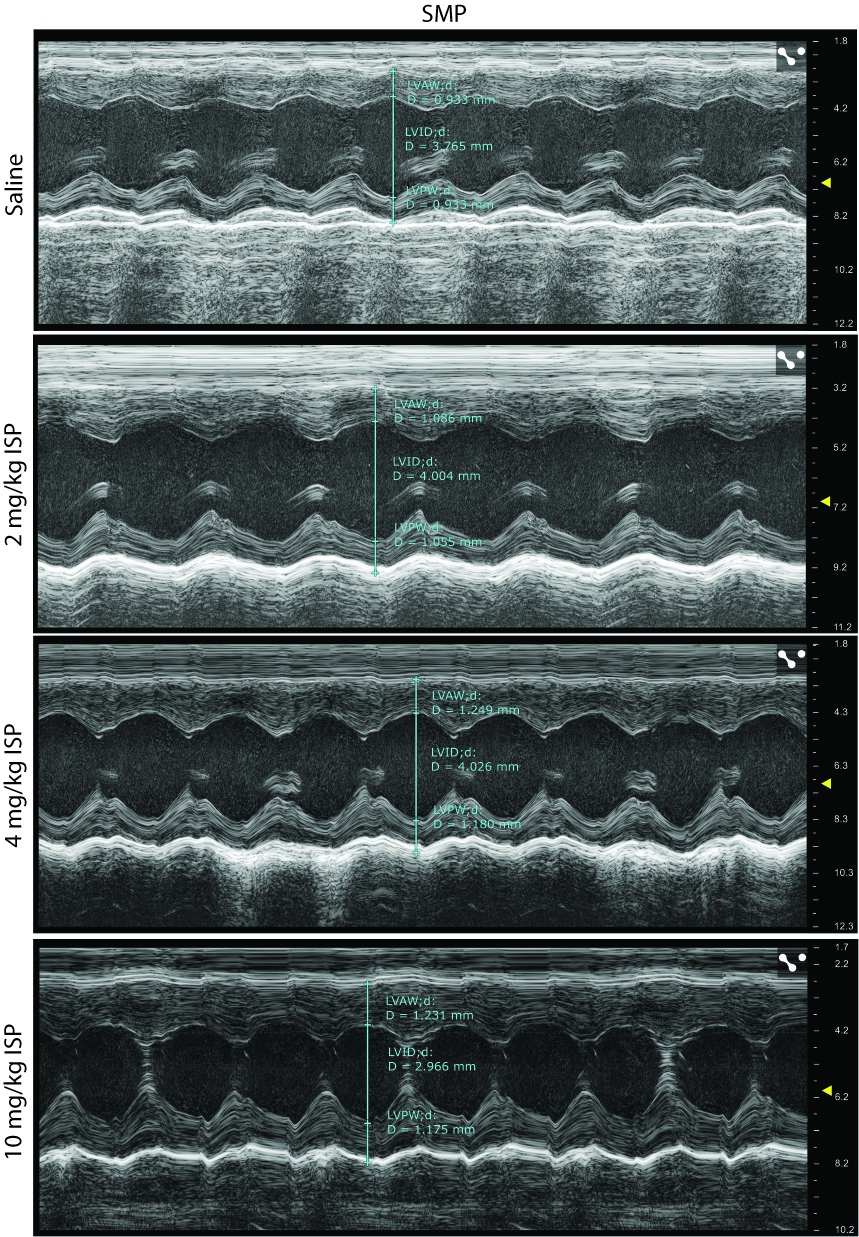

Supplement: S3 Fig — Echocardiography representative images for all SMP groups. (TIF) [file pone.0307467.s003.tif]
