# Supplementary material for: Isoproterenol induced cardiac hypertrophy: A comparison of three doses and two delivery methods in C57BL/6J mice
Source: PLoS One. 2024 Jul 22;19(7):e0307467. doi: 10.1371/journal.pone.0307467 (PMC11262646; doi:10.1371/journal.pone.0307467)
Supplement: S1 Table — Statistical tests used to compare between SQ and SMP groups treated with saline and all ISP doses showing mean and summary values. (PDF) [file pone.0307467.s004.pdf]

Supporting Table 1. Surface ECG statistics for comparison of mice in SQ vs SMP groups.

Figure	ANOVA Summary		Multiple comparison's test				
1A. Heart Rate	Ordinary one-way		Šídák's	Mean Diff.	95.00% CI of diff.	Summary	P Value
	F (DFn, DFd)	(7, 24)=13.04	SQ Saline vs. SMP Saline	3.9000	-69.78 to 77.58	ns	0.9998
	P value	**** <0.0001	SQ 2 vs. SMP 2	-119.3000	-193.0 to -45.65	***	0.0008
	R squared	0.7918	SQ 4 vs. SMP 4	-147.0000	-220.7 to -73.35	****	<0.0001
	Are SD different?	No	SQ 10 vs. SMP 10	-180.2000	-253.9 to -106.5	****	<0.0001
1B. RR Interval	Ordinary one-way		Šídák's	Mean Diff.	95.00% CI of diff.	Summary	P Value
	F (DFn, DFd)	(7, 24)=14.17	SQ Saline vs. SMP Saline	0.0000	-21.25 to 21.25	ns	>0.9999
	P value	**** <0.0001	SQ 2 vs. SMP 2	36.1000	14.85 to 57.35	***	0.0005
	R squared	0.8051	SQ 4 vs. SMP 4	43.2000	21.95 to 64.45	****	<0.0001
	Are SD different?	No	SQ 10 vs. SMP 10	54.1100	32.86 to 75.36	****	<0.0001
1C. P Duration	Ordinary one-way		Šídák's	Mean Diff.	95.00% CI of diff.	Summary	P Value
	F (DFn, DFd)	(7, 24)=0.8115	SQ Saline vs. SMP Saline	3.4770	-9.736 to 16.69	ns	0.9300
	P value	0.5865	SQ 2 vs. SMP 2	-6.2880	-19.50 to 6.925	ns	0.6153
	R squared	0.1914	SQ 4 vs. SMP 4	-0.8795	-14.09 to 12.33	ns	0.9996
	Are SD different?	No	SQ 10 vs. SMP 10	1.1370	-12.08 to 14.35	ns	0.9989
1D. PR Interval	Kruskal-Wallis		Dunn's	Mean Rank Diff.	Z	Summary	P Value
	Normal Distrubution?	No	SQ Saline vs. SMP Saline	0.5000	0.07538	ns	>0.9999
	P value	0.2656	SQ 2 vs. SMP 2	-12.5000	1.884	ns	0.2380
	Kruskal-Wallis statistic	8.824	SQ 4 vs. SMP 4	1.7500	0.2638	ns	>0.9999
	Are medians different?	No	SQ 10 vs. SMP 10	6.7500	1.018	ns	>0.9999
1E. QRS Interval	Kruskal-Wallis		Dunn's	Mean Rank Diff.	Z	Summary	P Value
	Normal Distrubution?	No	SQ Saline vs. SMP Saline	-4.2500	0.6407	ns	>0.9999
	P value	* 0.0405	SQ 2 vs. SMP 2	12.0000	1.809	ns	0.2818
	Kruskal-Wallis statistic	14.67	SQ 4 vs. SMP 4	16.7500	2.525	*	0.0463
	Are medians different?	Yes	SQ 10 vs. SMP 10	12.0000	1.809	ns	0.2818
1F. QT Interval	Kruskal-Wallis		Dunn's	Mean Rank Diff.	Z	Summary	P Value
	Normal Distrubution?	No	SQ Saline vs. SMP Saline	0.5000	0.08909	ns	>0.9999
	P value	0.0542	SQ 2 vs. SMP 2	5.5000	0.8001	ns	>0.9999
	Kruskal-Wallis statistic	13.83	SQ 4 vs. SMP 4	-3.3330	0.5499	ns	>0.9999
	Are medians different?	No	SQ 10 vs. SMP 10	0.2500	0.03637	ns	>0.9999
1G. P Amplitude	Kruskal-Wallis		Dunn's	Mean Rank Diff.	Z	Summary	P Value
	Normal Distrubution?	No	SQ Saline vs. SMP Saline	-1.5000	0.2261	ns	>0.9999
	P value	0.1416	SQ 2 vs. SMP 2	12.2500	1.847	ns	0.2591
	Kruskal-Wallis statistic	10.93	SQ 4 vs. SMP 4	13.2500	1.998	ns	0.1831

	Are medians different?	No	SQ 10 vs. SMP 10	1.5000	0.2261	ns	>0.9999
**1H. Q Amplitude**	Ordinary one-way		Šídák's	Mean Diff.	95.00% CI of diff.	Summary	P Value
	F (DFn, DFd)	(7, 24)=3.157	SQ Saline vs. SMP Saline	0.0163	-0.03940 to 0.07208	ns	0.9001
	P value	* 0.0164	SQ 2 vs. SMP 2	0.0042	-0.05154 to 0.05994	ns	0.9994
	R squared	0.4794	SQ 4 vs. SMP 4	-0.0274	-0.08316 to 0.02832	ns	0.5862
	Are SD different?	No	SQ 10 vs. SMP 10	-0.0482	-0.1039 to 0.007581	ns	0.1103
**1I. R Amplitude**	Ordinary one-way		Šídák's	Mean Diff.	95.00% CI of diff.	Summary	P Value
	F (DFn, DFd)	(7, 24)=11.72	SQ Saline vs. SMP Saline	0.1752	0.005737 to 0.3446	*	0.0407
	P value	**** <0.0001	SQ 2 vs. SMP 2	0.2929	0.1235 to 0.4623	***	0.0004
	R squared	0.7737	SQ 4 vs. SMP 4	0.2976	0.1281 to 0.4670	***	0.0003
	Are SD different?	No	SQ 10 vs. SMP 10	0.3267	0.1572 to 0.4961	***	0.0001
**1J. S Amplitude**	Ordinary one-way		Šídák's	Mean Diff.	95.00% CI of diff.	Summary	P Value
	F (DFn, DFd)	(7, 24)=6.484	SQ Saline vs. SMP Saline	0.03708	-0.1257 to0.1999	ns	0.9574
	P value	*** 0.0002	SQ 2 vs. SMP 2	-0.1625	-0.3253 to 0.0002545	ns	0.0505
	R squared	0.6541	SQ 4 vs. SMP 4	-0.2692	-0.4319 to -0.1064	***	0.0007
	Are SD different?	No	SQ 10 vs. SMP 10	-0.2386	-0.4014 to -0.07586	**	0.0024
