# Supplementary material for: Isoproterenol induced cardiac hypertrophy: A comparison of three doses and two delivery methods in C57BL/6J mice
Source: PLoS One. 2024 Jul 22;19(7):e0307467. doi: 10.1371/journal.pone.0307467 (PMC11262646; doi:10.1371/journal.pone.0307467)
Supplement: S2 Table — Statistical tests used to compare between SQ and SMP groups treated with saline and all ISP doses showing mean and summary values. (PDF) [file pone.0307467.s005.pdf]

Supporting Table 2. Heart function assessed via echocardiography comparison of mice in SQ vs SMP groups.

Figure	ANOVA Summary		Multiple comparison's test				
2A. % Ejection Fraction	Ordinary one-way		Šídák's	Mean Diff.	95.00% CI of diff.	Summary	P Value
	F (DFn, DFd)	(7, 55)=4.324	SQ Saline vs. SMP Saline	7.9000	-5.545 to 21.34	ns	0.4427
	P value	*** 0.0007	SQ 2 vs. SMP 2	-15.3000	-28.74 to -1.855	*	0.0195
	R squared	0.355	SQ 4 vs. SMP 4	-15.2300	-28.67 to -1.781	*	0.0203
	Are SD different?	No	SQ 10 vs. SMP 10	-14.7300	-28.65 to -0.8120	*	0.0339
2B. % Fractional Shortening	Kruskal-Wallis		Dunn's	Mean Rank Diff.	Z	Summary	P Value
	Normal Distrubution?	No	SQ Saline vs. SMP Saline	8.8750	0.9684	ns	>0.9999
	P value	** 0.0012	SQ 2 vs. SMP 2	-28.3100	3.089	**	0.0080
	Kruskal-Wallis statistic	23.95	SQ 4 vs. SMP 4	-21.8100	2.38	ns	0.0693
	Are medians different?	Yes	SQ 10 vs. SMP 10	-26.3400	2.776	*	0.0220
2C. LVAW Diastole	Ordinary one-way		Šídák's	Mean Diff.	95.00% CI of diff.	Summary	P Value
	F (DFn, DFd)	(7, 55)=6.424	SQ Saline vs. SMP Saline	-0.0400	-0.2211 to 0.1411	ns	0.9664
	P value	**** <0.0001	SQ 2 vs. SMP 2	-0.2400	-0.4211 to -0.05895	**	0.0048
	R squared	0.4498	SQ 4 vs. SMP 4	-0.2513	-0.4323 to -0.07020	**	0.0030
	Are SD different?	Yes	SQ 10 vs. SMP 10	-0.2118	-0.3992 to -0.02438	*	0.0207
2D. LVPW Diastole	Kruskal-Wallis		Dunn's	Mean Rank Diff.	Z	Summary	P Value
	Normal Distrubution?	No	SQ Saline vs. SMP Saline	-2.8130	0.3071	ns	>0.9999
	P value	**** <0.0001	SQ 2 vs. SMP 2	-34.5600	3.774	***	0.0006
	Kruskal-Wallis statistic	38.26	SQ 4 vs. SMP 4	-26.3800	2.88	*	0.0159
	Are medians different?	Yes	SQ 10 vs. SMP 10	-18.4400	1.945	ns	0.2071
2E. LVID Diastole	Ordinary one-way		Šídák's	Mean Diff.	95.00% CI of diff.	Summary	P Value
	F (DFn, DFd)	(7, 55)=1.580	SQ Saline vs. SMP Saline	-0.4681	-0.9905 to 0.05428	ns	0.0956
	P value	0.1610	SQ 2 vs. SMP 2	0.2375	-0.2849 to 0.7599	ns	0.6780
	R squared	0.1674	SQ 4 vs. SMP 4	0.3634	-0.1590 to 0.8858	ns	0.2798
	Are SD different?	No	SQ 10 vs. SMP 10	-0.0289	-0.5697 to 0.5118	ns	0.9999
2F. LVEDV	Ordinary one-way		Šídák's	Mean Diff.	95.00% CI of diff.	Summary	P Value
	F (DFn, DFd)	(7, 55)=1.606	SQ Saline vs. SMP Saline	-10.0400	-25.46 to 5.383	ns	0.3421
	P value	0.1531	SQ 2 vs. SMP 2	-4.1930	-16.61 to 11.23	ns	0.9306
	R squared	0.1697	SQ 4 vs. SMP 4	-8.0443	-23.46 to 7.376	ns	0.5582
	Are SD different?	No	SQ 10 vs. SMP 10	-10.7600	-26.72 to 5.198	ns	0.3084
2G-I. LV Mass	Ordinary one-way		Šídák's	Mean Diff.	95.00% CI of diff.	Summary	P Value
	F (DFn, DFd)	(7, 55)=9.574	SQ Saline vs. SMP Saline	-23.3300	-45.25 to -1.411	*	0.0326
	P value	**** <0.0001	SQ 2 vs. SMP 2	-35.0000	-56.92 to -13.08	***	0.0005
	R squared	0.5492	SQ 4 vs. SMP 4	-24.4300	-46.35 to -2.511	*	0.0230
	Are SD different?	No	SQ 10 vs. SMP 10	-31.4200	-54.11 to -8.729	**	0.0030
