# Supplementary material for: Isoproterenol induced cardiac hypertrophy: A comparison of three doses and two delivery methods in C57BL/6J mice
Source: PLoS One. 2024 Jul 22;19(7):e0307467. doi: 10.1371/journal.pone.0307467 (PMC11262646; doi:10.1371/journal.pone.0307467)
Supplement: S3 Table — Statistical tests used to compare between SQ and SMP groups treated with saline and all ISP doses showing mean and summary values. (PDF) [file pone.0307467.s006.pdf]

Supporting Table 3. Gene expression changes comparison of mice in SQ vs SMP groups.

Figure	ANOVA Summary		Multiple comparison's test				
**4A. *Acta2***	Ordinary one-way		Šídák's	Mean Diff.	95.00% CI of diff.	Summary	P Value
	F (DFn, DFd)	(7, 22)=50.39	SQ Saline vs. SMP Saline	-0.0035	-0.3837 to 0.3768	ns	>0.9999
	P value	**** <0.0001	SQ 2 vs. SMP 2	-1.1660	-1.547 to -0.7860	****	<0.0001
	R squared	0.9413	SQ 4 vs. SMP 4	-1.3190	-1.671 to -0.9673	****	<0.0001
	Are SD different?	No	SQ 10 vs. SMP 10	-1.0370	-1.389 to -0.6852	****	<0.0001
**4B. *Myh7***	Welch's		Dunnett's T3	Mean Diff.	95.00% CI of diff.	Summary	P Value
	Normal Distrubution?	Yes	SQ Saline vs. SMP Saline	-0.1149	-1.418 to 1.188	ns	0.9919
	W (DFn, DFd)	(7, 9.7)=3.905	SQ 2 vs. SMP 2	2.8660	0.9748 to 4.758	*	0.0119
	P value	* 0.0107	SQ 4 vs. SMP 4	6.3380	-8.239 to 20.91	ns	0.3335
	Are SD different?	Yes	SQ 10 vs. SMP 10	2.9930	0.7518 to 6.738	ns	0.0998
**4C. *Postn***	Welch's		Dunnett's T3	Mean Diff.	95.00% CI of diff.	Summary	P Value
	Normal Distrubution?	Yes	SQ Saline vs. SMP Saline	-0.0078	0.4591 to 0.4435	ns	>0.9999
	W (DFn, DFd)	(7, 9)=7.598	SQ 2 vs. SMP 2	3.2230	-3.821 to 10.27	ns	0.4328
	P value	** 0.0035	SQ 4 vs. SMP 4	0.7860	-5.657 to 7.229	ns	0.9743
	Are SD different?	Yes	SQ 10 vs. SMP 10	-1.4780	-4.928 to 1.971	ns	0.5245
**4D. *Nppa***	Welch's		Dunnett's T3	Mean Diff.	95.00% CI of diff.	Summary	P Value
	Normal Distrubution?	Yes	SQ Saline vs. SMP Saline	0.0560	-0.9010 to 1.013	ns	0.9964
	W (DFn, DFd)	(7, 8.2)=4.603	SQ 2 vs. SMP 2	2.6100	0.1686 to 5.051	*	0.0402
	P value	* 0.0229	SQ 4 vs. SMP 4	3.0590	-3.172 to 9.289	ns	0.2664
	Are SD different?	Yes	SQ 10 vs. SMP 10	1.4610	0.03918 to 2.883	*	0.0458
**4E. *Nppb***	Ordinary one-way		Šídák's	Mean Diff.	95.00% CI of diff.	Summary	P Value
	F (DFn, DFd)	(7, 22)=2.5	SQ Saline vs. SMP Saline	-0.0631	-0.8096 to 0.6834	ns	0.999
	P value	* 0.0474	SQ 2 vs. SMP 2	-0.0174	-0.8793 to 0.8446	ns	>0.9999
	R squared	0.443	SQ 4 vs. SMP 4	-0.1173	-0.8638 to 0.6292	ns	0.9887
	Are SD different?	No	SQ 10 vs. SMP 10	-0.2459	-0.9924 to 0.5006	ns	0.8535
